# ﻿Two new species of freshwater goby (Teleostei, Gobiidae) from the Upper Youshui River, Chongqing, China

**DOI:** 10.3897/zookeys.1210.128121

**Published:** 2024-08-22

**Authors:** Lingzhen Li, Chaoyang Li, Weihan Shao, Suxing Fu, Chaowei Zhou

**Affiliations:** 1 College of Fisheries, Southwest University, Chongqing, China; 2 Integrative Science Center of Germplasm Creation in Western China (CHONGQING) Science City, Key Laboratory of Freshwater Fish Reproduction and Development (Ministry of Education), Southwest University, Chongqing 400715, China; 3 Institute of Hydrobiology, Chinese Academy of Sciences, Wuhan, Hubei Province 430072, China

**Keywords:** China, fish taxonomy, Gobiidae, Gobionellinae, mitochondrial genome, Yuanjiang River Basin

## Abstract

Two previously unknown species of *Rhinogobius* have been discovered in the streams of the Upper Youshui River, within the Yuan River Basin, Xiushan County, Chongqing, China. These new species are named as *Rhinogobiussudoccidentalis* and *Rhinogobiuslithopolychroma*. Phylogenetic analysis based on mitochondrial genomes revealed that *R.sudoccidentalis* is genetically closest to *R.reticulatus*, while *R.lithopolychroma* shares the greatest genetic similarity with *R.leavelli*. Morphological distinctions allow for the clear differentiation of these species. *Rhinogobiussudoccidentalis***sp. nov.** is characterized by having VI–VII rays in the first dorsal fin and I, 8–9 rays in the second dorsal fin. The longitudinal scale series typically consists of 22–24 scales, while the transverse scale series comprises 7–8 scales. Notably, the predorsal scale series is absent and the total vertebrae count is 12+17=29. *Rhinogobiuslithopolychroma***sp. nov.** can be distinguished from other species by the presence of 13–15 rays on the pectoral fin. Its longitudinal scale series ranges from 30 to 33 scales, with no scales in the predorsal area. The total vertebral count is 30, with 12 precaudal and 18 caudal vertebrae. The head and body of this species are light gray with irregular orange markings on the cheeks and opercle. Through morphological and molecular analyses, it has been confirmed that *R.lithopolychroma* and *R.sudoccidentalis* represent novel species within the *Rhinogobius* genus.

## ﻿Introduction

The genus *Rhinogobius*, belonging to the subfamily Gobionellinae within the family Gobiidae, is widely distributed across East and Southeast Asia. First described by Gill in 1859, with *Rhinogobiussimilis* Gill, 1859 as the type species, this genus is known for its high species richness. Over 92 valid species have been described, with an increasing number of new species being discovered. In recent years, several new species of *Rhinogobius* have been found in China, including *R.houheensis* Kunyuan et al., 2020, *R.coccinella* Endruweit, 2018, *R.maculagenys*Wu et al., 2018, *R.maxillivirgatus*Xia et al., 2018, *R.nanophyllum* Endruweit, 2018, *R.wuyanlingensis*Huang et al., 2016, *R.niger*Huang et al., 2016, *R.immaculatus* Li et al., 2018, *R.lintongyanensis* Chen et al., 2022 and *R.lianchengensis* Wang & Chen, 2022. To date, a total of 47 species of *Rhinogobius* have been recorded in China (Chen et al. 2022a) . The significant diversity of *Rhinogobius* species in China suggests that the overall species diversity within this genus may be underestimated. Notably, the recent discoveries of *Rhinogobius* species have been concentrated in East China, with fewer new species found in other regions.

The Upper Yuanjiang River Basin benefits from a favorable climate and encompasses numerous stream habitats within its mountainous areas. The biodiversity in Xuan’en and Fanjingshan, traversed by the Upper Yuanjiang River Basin, is exceptionally high and potentially serves as a glacial refuge (Fei et al. 2017). Consequently, it is inferred that the biodiversity in other regions of the Upper Yuanjiang River Basin, particularly within its stream habitats, may have been underestimated.

During surveys conducted between June 2023 and January 2024 in the streams of the Upper Youshui River within the Yuanjiang River Basin in Chongqing, two species of *Rhinogobius* were discovered. Historically, only *R.similis* and *Rhinogobiuscliffordpopei* (Nichols, 1925) were documented in the Yuanjiang River Basin in Chongqing, with these species primarily adapted to lake and reservoir environments (Wu et al. 2008; Suzuki et al. 2016). In contrast, the newly discovered species exclusively inhabit streams and are characterized by large eggs, unlike *R.similis* and *R.cliffordpopei*, which produce small eggs (Li 2011). The Upper Youshui River features a diverse stream ecosystem where species distribution is influenced by factors such as substrate composition, temperature, and current velocity. This study delves into the habitat of *Rhinogobius* in the Upper Youshui River to explore the habitat segregation of *Rhinogobius*, building upon previous research concerning the ecological preferences of *Rhinogobius* species (Sone et al. 2001; Ito et al. 2006).

## ﻿Materials and methods

### ﻿Samples

**Figure 1.**
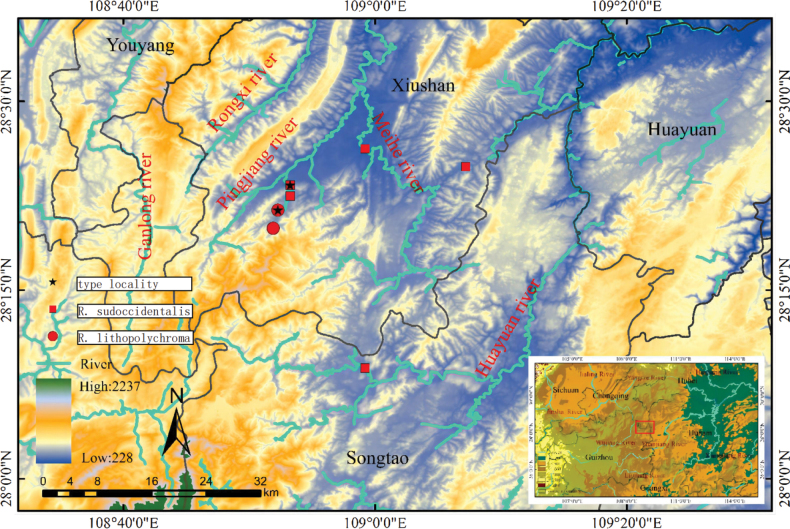
Map of the distribution of *Rhinogobiussudoccidentalis* sp. nov. and *Rhinogobiuslithopolychroma* sp. nov. in Upper Youshui River, with locations in southwest China shown in the lower right corner. Maps were prepared using ArcMap 10.8.

A total of 44 specimens were collected from Chongqing Municipality and Guizhou Province (Fig. 1) using a hand net. All specimens were preserved in 75% ethanol and are stored at Southwest University in Rongchang District, Chongqing, China.

### ﻿Morphometrics and meristic methods

Morphological measurements were primarily based on a previous study (Wu et al. 2008). Data were collected from the left side of each fish using vernier calipers, measuring 27 traits to the nearest 0.1 mm. Measurements included the first dorsal fin, second dorsal fin, pectoral fin, anal fin, longitudinal scales, transverse scales, and predorsal scales. Abbreviations for the cephalic sensory pore system followed Chen and Kottelat (2005). The pattern of interdigitation of the dorsal-fin pterygiophores and neural spines (P-V) was observed from radiographs. The P-V method and vertebral counting were expressed using a specific formula to describe the goby’s interdigitation pattern of dorsal-fin pterygiophores and neural spines (Akihito et al. 1984). For example, in the formula (P-V) 3/II II I I I 0/9: (P-V) stands for dorsal-fin pterygiophores and neural spines; “3” indicates that three neural spines are anterior to the first pterygiophore; “II II I I” indicates there are 2 pterygiophores between the neural spine of the 3^rd^ and 4^th^ vertebrae; 2 between the neural spine of the 4^th^ and 5^th^ vertebrae; 1 between the neural spine of the 5^th^ and 6^th^ vertebrae; and 1 between the neural spine of the 5^th^ and 6^th^ vertebrae; “0” indicates no pterygiophore between the neural spines of the 7^th^ and 8^th^ vertebra; “9” indicates that the first pterygiophore of the 1^st^ ray of the 2^nd^ dorsal fin is inserted above the 9^th^ vertebral body. Color in life was described based on samples and photographs taken in fish tanks.

### ﻿DNA sequencing and phylogenetic analysis

Four specimens were used for DNA barcoding. Total DNA was extracted from the caudal fin following Maeda et al. (2021a) and Wanghe et al. (2020). Briefly, single-stranded circular DNA molecules were amplified into a DNB (DNA Nanoball) containing more than 300 copies via rolling circle replication. These DNBs were then applied to mesh pores on the chip using high-density DNA nano-chip technology. Sequencing was performed by cPAS. Identification of complete mitochondrial genomes from assembled contigs was achieved through two criteria: 1) comparison with the complete mitochondrial genome of *Stiphodonalcedo* Maeda, Mukai, & Tachihara, 2011 (accession: AB613000.1) (BLASTN e-value ≤ 1e-100), and 2) confirmation that 100 bp of both the head and tail DNA sequences of a contig were identical, indicating that the sequence was circular. Complete mitochondrial genomes were aligned using MAFFT v.7.244 (Katoh and Standley 2013). The obtained mitochondrial gene was compared with *Rhinogobiuswuyanlingensis* Yang, Wu & Chen, 2008 (accession: NC_062781.1), confirming identical sequences at the head and tail DNA regions, indicative of circularity. Aligned mitochondrial genomes underwent phylogenetic analysis using maximum likelihood (ML) methods with RAxML v. 8.2.3 (Stamatakis 2014), incorporating mitochondrial gene data from the GenBank library (Table 1). Outgroup specimens were analyzed using *Tridentigerkuroiwae* Jordan & Tanaka, 1927 (accessions: LC653489.1 and LC65349.1). The aligned mitochondrial genomes from this study have been deposited in the GenBank library under accession numbers SRR28284917-SRR28284920.

**Table 1.** List of accession numbers and sequence length of mitochondrial genome sequences in this study.

	Accession number	Length of sequence (bp)	Remarks
*Rhinogobiusestrellae*	LC648292	16682	Maeda et al. (2021b)
*Rhinogobiusestrellae*	LC648294	16504	Maeda et al. (2021b)
*Rhinogobiusestrellae*	LC648295	16505	Maeda et al. (2021b)
*Rhinogobiusestrellae*	LC648296	16504	Maeda et al. (2021b)
*Rhinogobiustandikan*	LC648297	16691	Maeda et al. (2021b)
*Rhinogobiustandikan*	LC648298	16690	Maeda et al. (2021b)
*Rhinogobiustandikan*	LC648299	16918	Maeda et al. (2021b)
*Rhinogobiustandikan*	LC648300	16690	Maeda et al. (2021b)
*Rhinogobiussimilis*	LC648303	16499	Maeda et al. (2021b)
*Rhinogobiussimilis*	LC648304	16499	Maeda et al. (2021b)
*Rhinogobiusformosanus*	MT363639	16500	Yang et al. (2020)
*Rhinogobiusformosanus*	MN549279	16502	Genbank
*Rhinogobiusszechuanensis*	OM617727	16492	Liu WZ et al. (2023)
*Rhinogobiusleavelli*	MH729000	16499	Zhang and Shen (2019)
*Rhinogobiusdavidi*	OM617724	16627	Song et al. (2023)
*Rhinogobiusrubromaculatus*	KU674802	16503	Genbank
*Rhinogobiusflumineus*	LC648305	16504	Maeda et al. (2021b)
*Rhinogobiusflumineus*	LC648306	16503	Maeda et al. (2021b)
*Rhinogobiusyaima*	LC648307	16500	Maeda et al. (2021b)
*Rhinogobiusyaima*	LC648308	16500	Maeda et al. (2021b)
*Rhinogobiusyonezawai*	LC648309	16500	Maeda et al. (2021b)
*Rhinogobiusyonezawai*	LC648310	16500	Maeda et al. (2021b)
*Rhinogobiusnagoyae*	LC648315	16498	Maeda et al. (2021b)
*Rhinogobius* sp. MO	LC648314	16499	Maeda et al. (2021b)
*Rhinogobiusbrunneus*	LC648311	16500	Maeda et al. (2021b)
*Rhinogobiusbrunneus*	LC648312	16500	Maeda et al. (2021b)
*Rhinogobiuswuyiensis*	OM678441	16502	Chen XJ et al. (2022b)
*Rhinogobiuslentiginis*	OM617725	16633	Chen XJ et al. (2022b)
*Rhinogobiusniger*	OM791349	16496	Genbank
*Rhinogobiusmaculagenys*	OK545540	16500	Hu J et al. (2023)
*Rhinogobiusshennongensis*	OM961050	16500	Genbank
*Rhinogobiuscliffordpopei*	KX898434	16511	Genbank
*Rhinogobiuscliffordpopei*	KP694000	16529	Genbank
*Rhinogobiuscliffordpopei*	KT357638	16525	Genbank
*Rhinogobiusduospilus*	MH127918	16496	Tan et al. (2020)
*Rhinogobiusfilamentosus*	OM678440	16510	Chen XJ et al. (2022b)
*Rhinogobiuswuyanlingensis*	OM617722	16491	Song et al. (2022)
*Rhinogobiuswuyanlingensis*	OM961051	16491	Genbank
*Rhinogobius* sp. Xiushan	SRR28284919	16486	Collected in Xiushan, Chongqing
*Rhinogobiuslithopolychroma*	SRR28284920	16493	Collected in Xiushan, Chongqing
*Rhinogobiussudoccidentalis*	SRR28284918	16480	Collected in Xiushan, Chongqing
*Rhinogobiusreticulatus*	SRR28284917	16497	Collected in Fuzhou, Fujian Province
*Tridentigerkuroiwae*	LC653489	16501	Maeda et al. (2021b)
*Tridentigerkuroiwae*	LC653490	16501	Maeda et al. (2021b)

## ﻿Results

### ﻿Morphological analyses

#### ﻿ Rhinogobius sudoccidentalis sp. nov.

Taxon classificationAnimaliaPerciformesGobiidae

6F20D029-06DD-54BD-8900-8DD3FE17B858

https://zoobank.org/975F33AC-F810-4F32-8D57-26A583D924BB

**Figure 2.**
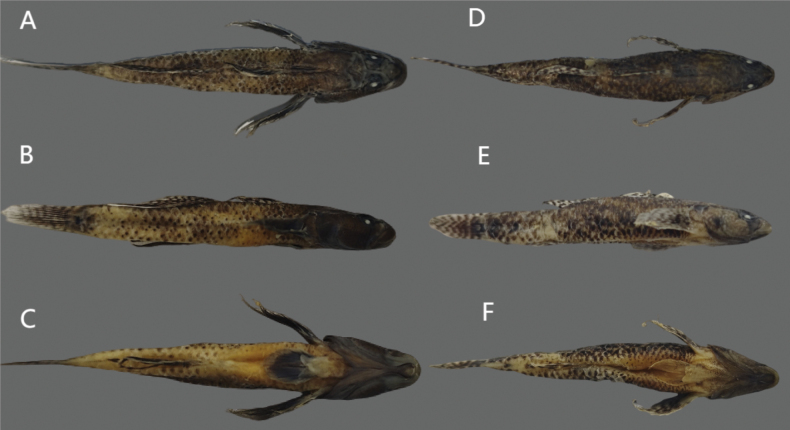
Dorsal (**A**), lateral (**B**), and ventral (**C**) views of preserved holotype of *Rhinogobiussudoccidentalis* sp. nov. (RS20230001 male) and dorsal (**D**), lateral (**E**), and ventral (**F**) views of preserved paratype of *Rhinogobiussudoccidentalis* sp. nov (RS20230101 female).

**Figure 3.**
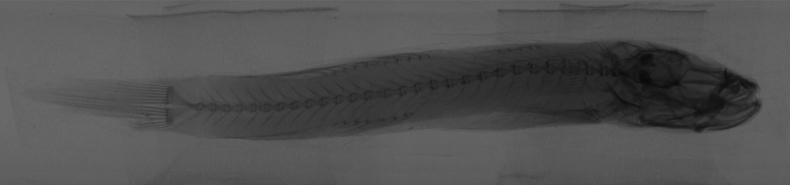
The skeletal system of *R.sudoccidentalis* sp. nov. Radiograph graphs of the whole body for paratype RS20230102, male.

**Figure 4.**
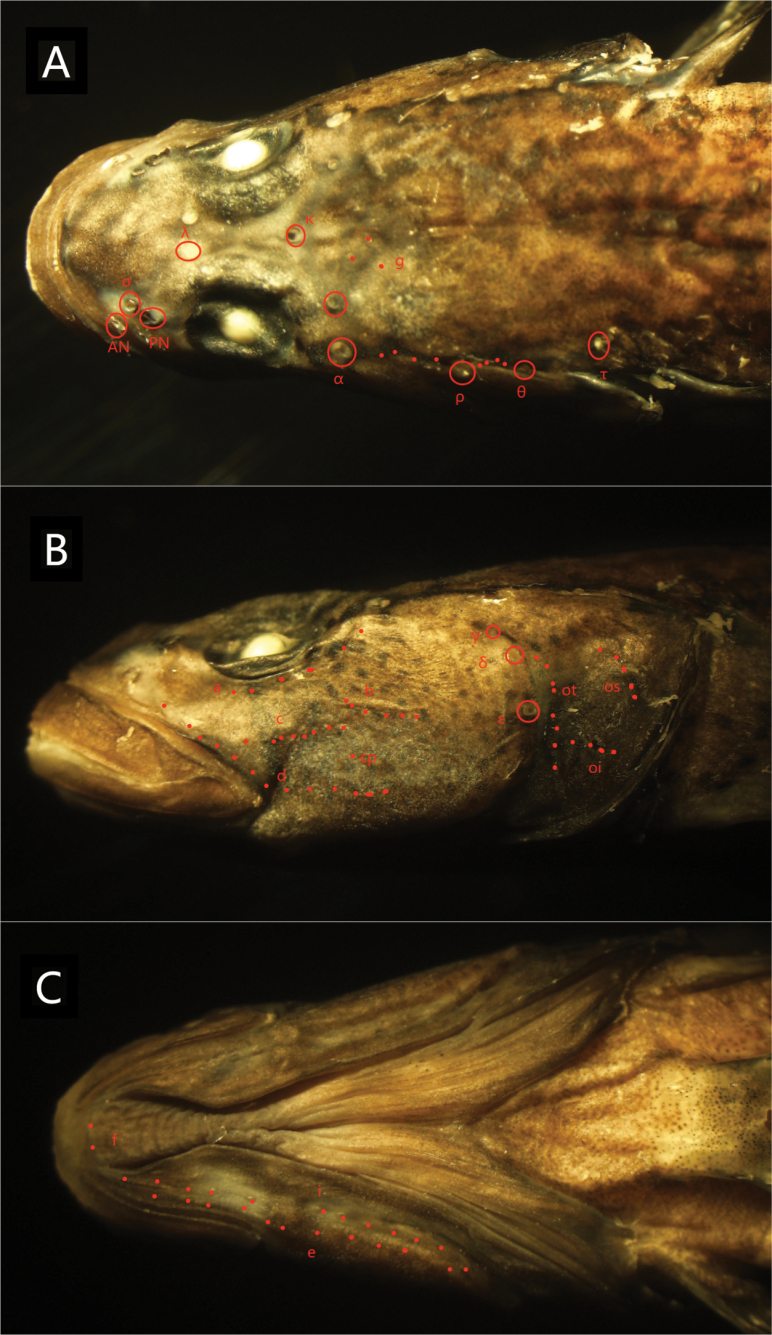
Dorsal (**A**), lateral (**B**), and ventral (**C**) views of the head of the preserved holotype of *R.sudoccidentalis* sp. nov. Red circles indicate sensory canal pores; red dots represent sensory papillae. Abbreviations: AN, anterior nare pore; PN, posterior nare pore

**Figure 5.**
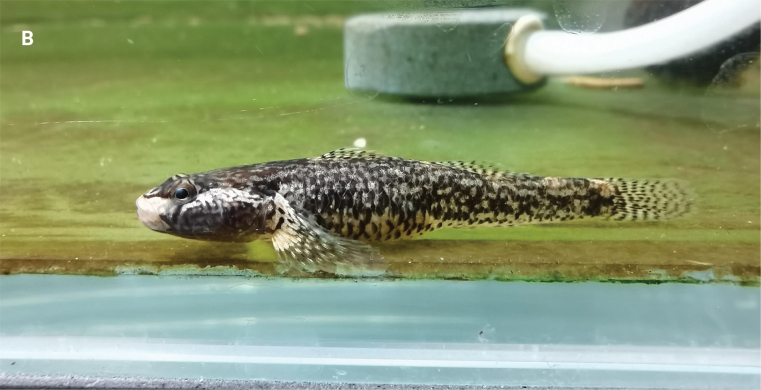
Photographs of *R.sudoccidentalis* sp. nov. captured underwater in a tank **A** male **B** female. Photographed by Mr Zhi.

**Figure 6.**
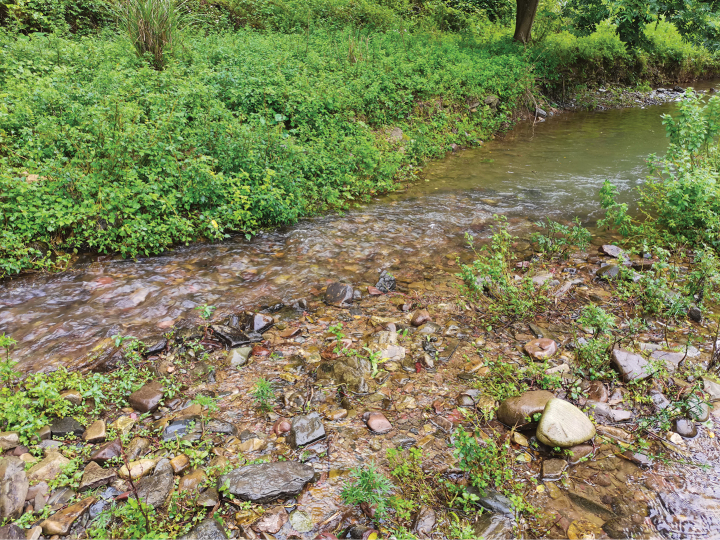
Stream environment in Xiushan, Chongqing where *R.sudoccidentalis* sp. nov. was collected.

**Figure 7.**
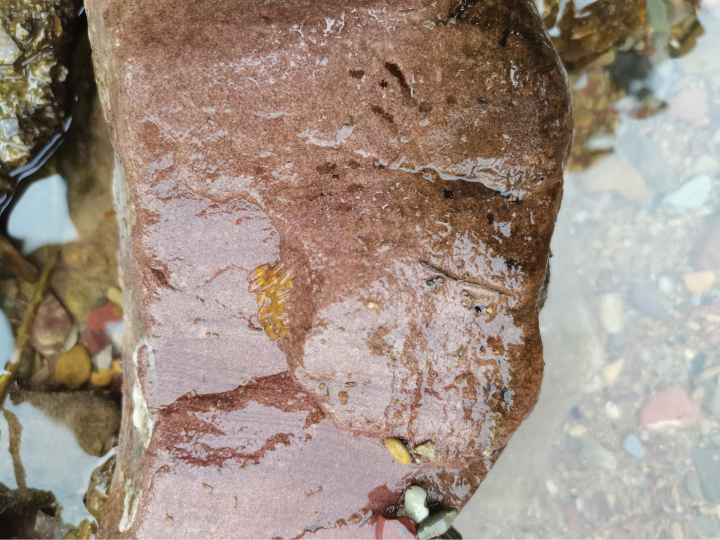
Eggs of *R.sudoccidentalis* sp. nov. at the type locality.

Table 2 , Figs 2 , 3 , 4 , 5 , 6 , 7

##### Type materials.

***Holotype*.** China • 1 ♂; Chongqing City, Xiushan County; 28°23'23"N, 108°53'16"E; 1 July. 2023; Lingzhen Li & Chaoyang Li leg.; RS20230001.

***Paratypes*.** China - Chongqing City • 7 ♂♂, 3 ♀♀; Xiushan County; 28°23'23"N, 108°53'16"E; 1 July. 2023; Lingzhen Li & Chaoyang Li leg.; RS20230101 to 20230110. • 4 ♂♂ ; Xiushan County; 28°26'17"N, 108°59'12"E; 1 July. 2023; Lingzhen Li & Chaoyang Li leg.; RS20230111 to 20230114. • 1 ♂ , 1 ♀ ; Xiushan County; 28°24'51"N, 109°7'13"E ; 3 July. 2023; Lingzhen Li & Chaoyang Li leg.; RS20230115, 20230116. • 1 ♂ , 2 ♀♀ ; Xiushan County; 28°22'30"N, 108°53'18"E; 4 July. 2023; Lingzhen Li & Chaoyang Li leg.; RS20230118, 20230120. - Guizhou Province • 1 ♂ ; Tongren City; 28°8'50"N, 108°59'13"E; 3 July. 2023; Lingzhen Li & Chaoyang Li leg.; RS20230117.

##### Diagnosis.

*Rhinogobiussudoccidentalis* can be distinguished from other species in the genus by the following characteristics: it possesses VI–VII rays in the first dorsal fin and I, 8–9 rays in the second dorsal fin. The longitudinal scale series typically consists of 22–24 scales (most commonly 23), while the transverse scale series typically comprises 7–8 scales (most commonly 8). The predorsal scale series is absent. The total number of vertebrae counts is 12+17=29. Additionally, it features a black line stripe beneath the eye that extends to the mandible. Morphometrics Reference Table 2.

**Table 2.** Morphometrics of the types of *R.sudoccidentalis* expressed as a percentage of standard length.

Variable	Holotype	Paratypes	
Sex	males	males (*N* = 14)	Females (*N* = 6)
Morphometry			
Standard length (mm)	33.1	33.1–40.6(36.5)	30.2–36.5(32.1)
Head length (mm)	8.9	8.9–11.5(10.3)	7.3–9.9(8.1)
Percent standard length (%)			
Head length	26.9	26.5–30.3(28.4)	23.7–27.1(25.2)
Predorsal length	37.8	31.7–43.1(37.4)	34.5–39.0(36.9)
Snout to second dorsal fin origin	53.8	53.6–59.2(56.2)	57.1–59.4(58.4)
Snout to anal fin origin	59.5	55.4–64.9(58.9)	59.3–64.7(62.7)
Snout to anus	54.1	51.2–56.9(53.5)	50.5–59.0(56.0)
Pre pelvic length	28.7	28.7–35.7(31.3)	28.8–33.7(30.6)
Caudal peduncle length	26.9	21.8–29.2(25.7)	17.3–27.5(23.2)
Caudal peduncle depth	8.8	8.2–10.5(9.2)	8.0–11.9(9.5)
First dorsal-fin base	8.5	8.5–17.3(12.8)	8.6–13.5(11.2)
Second dorsal-fin base	16.3	13.1–19.9(16.9)	14.6–19.6(16.1)
anal fin base	14.2	8.5–14.3(11.7)	8.7–11.7(10.0)
Caudal fin length	20.8	18.4–26.1(22.1)	13.5–23.8(18.5)
Pectoral fin length	20.2	19.6–24.1(21.8)	16.6–21.0(18.7)
Pelvic fin length	14.5	13.5–19.2(15.7)	12.5–18.1(15.8)
Body depth of pelvic fin origin	9.1	9.1–14.2(11.5)	9.8–12.9(11.7)
Body depth of anal fin origin	9.4	8.3–13.0(10.4)	9.3–11.5(10.6)
Pelvic fin origin to anus	26.9	22.0–27.2(25.2)	25.6–30.8(27.4)
Head depth	9.7	9.7–12.2(11.0)	9.6–12.9(11.0)
Percent head length (%)			
Snout length	31.5	22.8–37.4(30.6)	19.2–31.6(25.5)
Eye diameter	14.6	11.3–19.3(14.2)	10.4–16.5(12.0)
Cheek depth	56.2	20.7–32.2(25.5)	21.2–29.1(24.1)
Postorbital length	55.1	43.1–60.4(51.7)	49.5–58.9(54.4)
Lower jaw length	31.5	27.9–48.7(38.7)	24.2–37.7(31.0)
Interorbital width	22.5	11.9–24.0(20.8)	12.1–19.5(16.3)
Head width in maximum	51.7	45.5–61.7(52.7)	50.5–65.8(58.0)

##### Description.

***Fins***: The fins display distinct features: the first dorsal fin typically bears VI rays (18) or VII rays (2), while the second dorsal fin exhibits either I, 8 rays (2) or I, 9 rays (18). The 3^rd^ or 4^th^ spine of the first dorsal fin is the longest and lacks filamentous. In males, the depressed first dorsal fin extends to the base of the 1^st^ or 2^nd^ branched ray of the second dorsal fin; in females, it reaches only the base of the second dorsal fin anteriorly. The anal fin has I, 6 rays (1) or I, 7 rays (19), originating at a vertical line between the 2^nd^ and 3^rd^ branched soft ray of the second dorsal fin. The pectoral fin typically has 14 rays (2) or 15 rays (18) and is broad. In males, the rear tip of the pectoral fin aligns parallel to the anus, a feature absent in females.

***Scales***: The body is covered with ctenoid scales, with enlarged mid-trunk scales. The anterior predorsal area lacks scales, while the posterior occipital region is adorned with cycloid scales. The belly is covered with small cycloid scales. The longitudinal scale series ranges from 22 to 24 (mode: 23), and the transverse scale series ranges from 7 to 8 (mode: 8). No scales are present in the predorsal area.

***Head canals***: Pores σ are located between the anterior and posterior nares. The anterior interorbital sections of oculoscapular canal are separated, featuring paired pore λ. A single pore κ is situated in the posterior region, with ω present near posterior edge of eyes. There is an absence of ω1. The lateral section of anterior oculoscapular canal exhibits pores α and terminal pore ρ. The posterior oculoscapular canal ends with two terminal pores θ and τ. Preopercular canals are presented, featuring pores ε, γ, and δ.

***Sensory papillae***: Row a extends anteriorly to just before the middle of the eye. Row b is oblique and reaches forward to the posterior margin of the eyes. Rows c and d are longer, extending behind the orbit, with Row cp positioned between Rows c and d. Row f is paired. Opercular papillae include Rows ot, oi, and os, with oi nearly reaching ot.

***Vertebrae***: The total vertebrae count is 12 + 17 = 29 (*N* = 5), with a (P–V) pattern of 3/II II I I 0/9 (*N* = 5).

***Coloration of preserved specimens***: In males, the head and body of *R.sudoccidentalis* exhibit a yellowish-brown color. There are paired brown stripes on the snout converging at the tip, while the cheeks and opercle are adorned with small black spots. A black stripe extends from under the eye to the mandible. The ventral side displays dens coverage of small black spots. The membrane of the first dorsal fin is gray, the second dorsal fin has a transparent membrane with dense black mottling, and the anal fin exhibits a black membrane. The pectoral fin is transparent. In females, the head and body are yellowish, with a single black diagonal line below each eye. Irregular black patches are present on the ventral side, and both the dorsal and anal fins are transparent.

***Color in life***: In males, the head and body of the *R.sudoccidentalis* are creamy white. There are paired reddish-brown stripes on the snout meeting at the tip, and the cheeks and opercle feature small black spots. A black stripe extends from under the eye to the mandible. The ventral side is densely covered with small orange spots. The membrane of the first dorsal fin is red with a blue mottling pattern between the 1^st^ and 2^nd^ spinous rays. The second dorsal fin has a transparent membrane with dense black mottling and a white outer edge. The anal fin exhibits a white margin with reddish dots on the ventral part of the reddish membrane. The pectoral fin is transparent, with a milky white basal portion. In females, the head and body are yellowish, with paired brown stripes on the snout meeting at the tip. There are single black diagonal lines below the eyes, and irregular black patches on the ventral side. Both the dorsal and anal fin are transparent, and the pectoral fin is transparent with a milky white basal portio.

##### Distribution and habitat.

*Rhinogobiussudoccidentalis* was initially discovered in a small stream in Xiushan, Chongqing, where it predominantly inhabits areas characterized by large cobblestone substrates and slow-flowing water at depths ranging from approximately 30 to 50 cm. Additionally, small populations of this species were also observed in Tongren, Guizhou Province. In the Xiushan area, *R.sudoccidentalis* is the dominant fish species, utilizing the cobblestone bottom as an egg deposition site, with eggs characterized as large (size 1.6–2.1 mm). During periods of high water levels in the creek, individuals aggregate near the shore to seek refuge from the rapids.

##### Etymology.

This species, discovered in Chongqing and Guizhou Province in the southwestern region of China, has been named *R.sudoccidentalis*. The Latin roots “sud” meaning “south” and “occidentalis” meaning “western” combine to signify “southwestern”. The suggested Chinese name for this species is 西南吻虾虎鱼.

#### ﻿ Rhinogobius lithopolychroma sp. nov.

Taxon classificationAnimaliaPerciformesGobiidae

FB9AABE2-DE19-5C72-BC97-39ACA96ABDF9

https://zoobank.org/C1F210C4-1623-4B50-BB2A-F9DBAD7F197A

**Figure 8.**
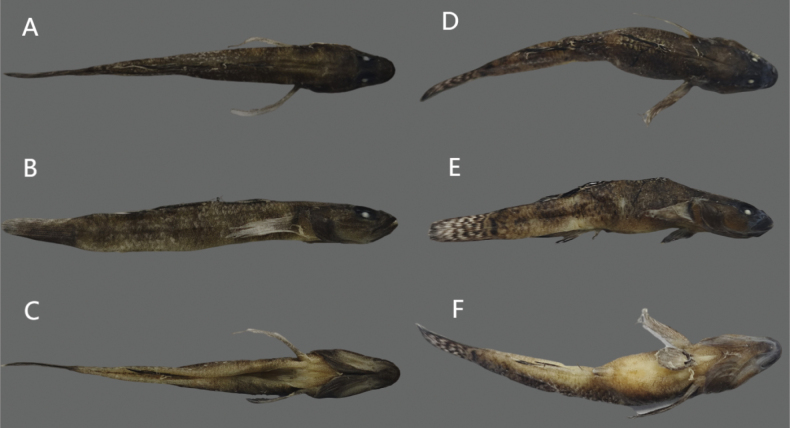
Dorsal (**A**), lateral (**B**), and ventral (**C**) views of preserved holotype of *R.lithopolychroma* sp. nov. (RL20230001 male) and dorsal (**D**), lateral (**E**), and ventral (**F**) views of preserved paratype of *R.lithopolychroma* sp. nov. (RL20230101 female).

**Figure 9.**
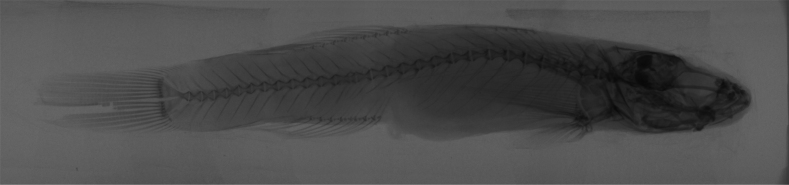
The skeletal system of *R.lithopolychroma* sp. nov. Radiograph graphs of the whole body for paratype RL20230201, females.

**Figure 10.**
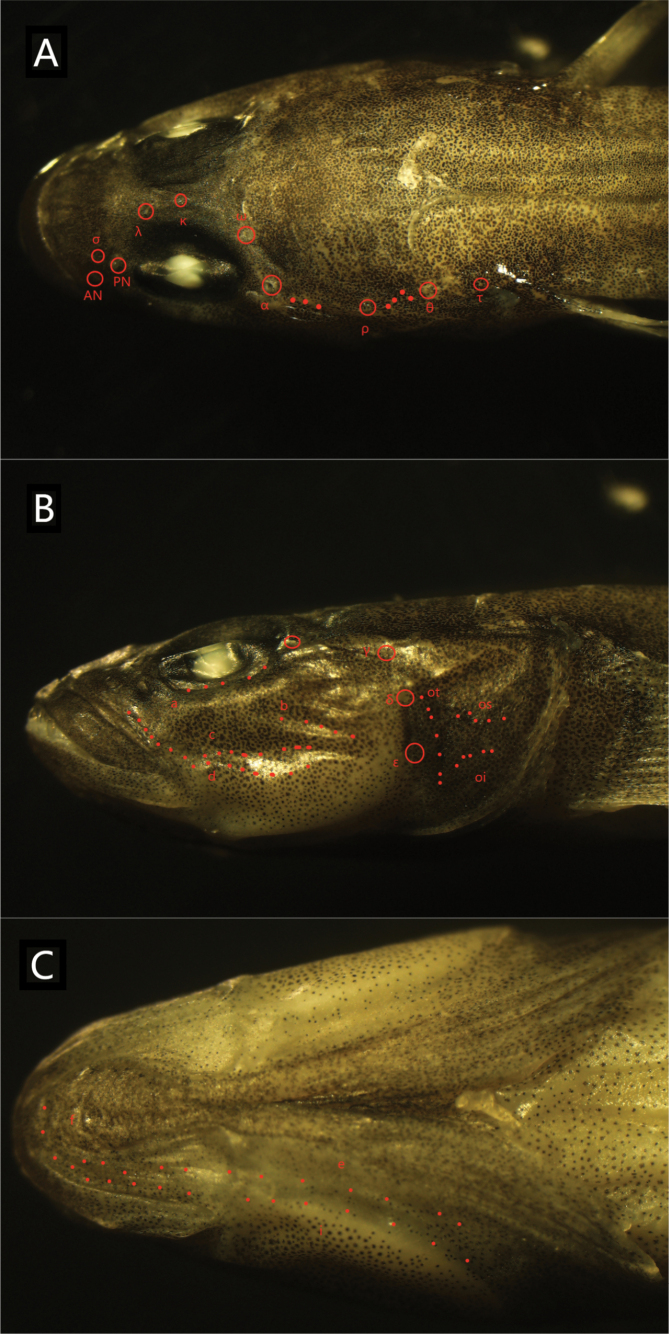
Dorsal (**A**), lateral (**B**), and ventral (**C**) views of the head of the preserved holotype of *R.lithopolychroma* sp. nov. Red circles indicate sensory canal pores; red dots represent sensory papillae. Abbreviations: AN, anterior nare pore; PN, posterior nare pore.

**Figure 11.**
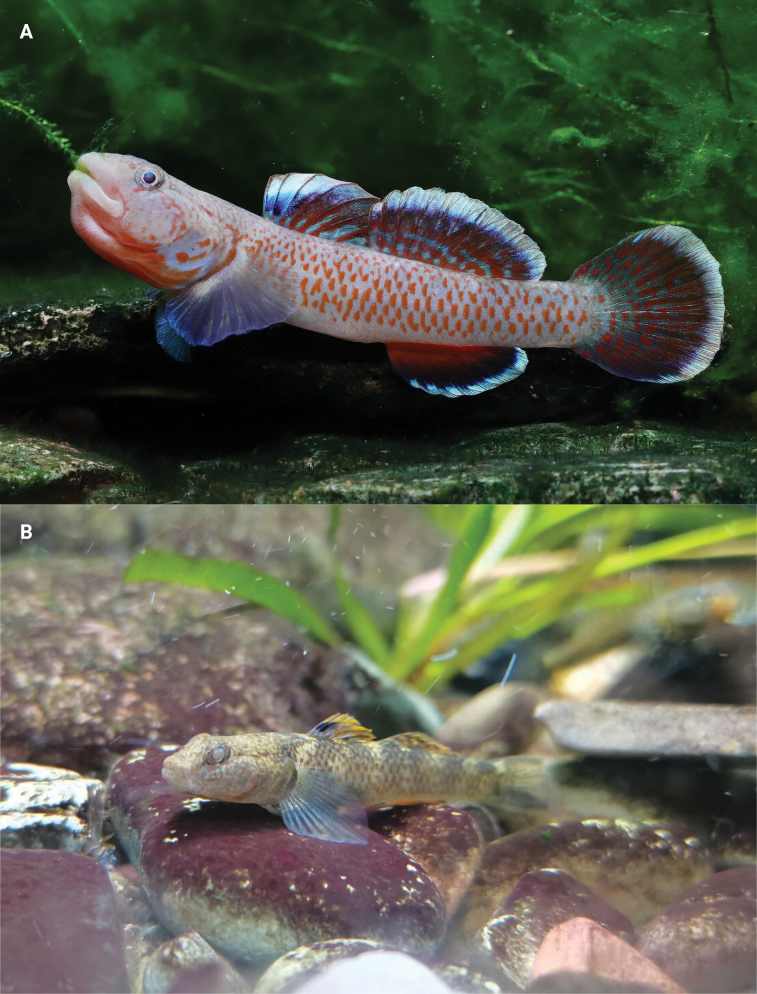
Photographs of *R.lithopolychroma* captured underwater in a tank **A** male and **B** female. Photographed by Mr Zhi.

**Figure 13.**
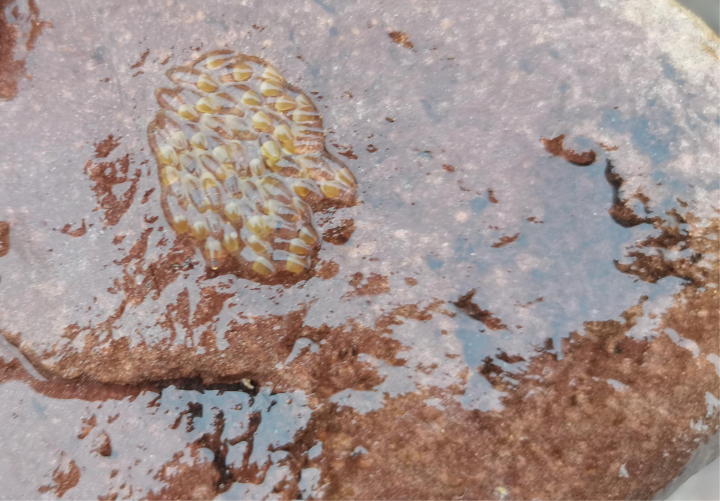
Eggs of *R.lithopolychroma* sp. nov. at the type locality.

Table 3 , Figs 8 , 9 , 10 , 11 , 12 , 13

##### Type materials.

***Holotype*.** China • 1 ♂; Chongqing City, Xiushan County; 28°21'21"N, 108°52'16"E; 2 July. 2023; Lingzhen Li & Chaoyang Li leg.; RL20230001.

***Paratypes*.** China • Chongqing City • 6 ♂♂, 4 ♀♀; Xiushan County; 28°21'21"N, 108°52'16"E; 2 July. 2023; Lingzhen Li & Chaoyang Li leg.; RL20230101 to 20230110. • 11 ♂♂, 1 ♀; Xiushan County; 28°19'56"N, 108°52'17"E; 4 July. 2023; Lingzhen Li & Chaoyang Li leg.; RL20230111 to 20230122.

##### Diagnosis.

*Rhinogobiuslithopolychroma* can be distinguished from other species in the *Rhinogobius* by the following characteristics: It typically possesses 13–15 rays on the pectoral fin. The longitudinal scale series count ranges from 30 to 33, with the predorsal area lacking scales. The total vertebrae count is 30, comprising 12 precaudal and 18 caudal vertebrae. The head and body of this species are light gray, adorned with irregular orange markings on the cheeks and opercle. Morphometrics Reference Table 3.

**Table 3.** Morphometrics of the types of *R.lithopolychroma* expressed as a percentage of standard length.

Variable	Holotype	Paratypes	
Sex	males	males (*N* = 17)	Females (*N* = 5)
Morphometry			
Standard length (mm)	28.2	28.2–38.8(31.1)	27.5–36.4(33.6)
Head length (mm)	9.5	7.9–11.6(9.6)	7.9–10.7(9.7)
Percent standard length (%)			
Head length	33.7	25.8–33.7(28.9)	25.8–30.3(28.8)
Predorsal length	36.5	28.2–43.4(37.2)	32.4–41.6(37.1)
Snout to second dorsal fin origin	58.2	42.0–58.5(54.6)	54.2–62.0(58.9)
Snout to anal fin origin	66.7	56.7–66.7(61.8)	63.5–66.9(65.1)
Snout to anus	56.4	51.5–57.2(55.2)	55.6–61.2(58.5)
Pre pelvic length	31.9	26.2–34.8(30.5)	28.3–34.9(31.6)
Caudal peduncle length	18.8	18.8–23.1(21.1)	18.4–24.1(21.4)
Caudal peduncle depth	10.6	9.0–12.2(10.6)	9.5–11.4(10.6)
First dorsal-fin base	13.5	10.3–15.2(13.0)	9.9–14.2(11.9)
Second dorsal-fin base	22.3	16.1–22.8(19.1)	14.5–21.2(16.6)
anal fin base	15.2	10.8–15.8(13.8)	10.4–15.7(12.2)
Caudal fin length	28.0	15.4–28.0(22.7)	17.8–23.4(20.3)
Pectoral fin length	25.5	19.3–26.7(22.4)	20.5–21.2(21.0)
Pelvic fin length	11.7	9.6–13.8(11.3)	9.9–12.7(11.5)
Body depth of pelvic fin origin	11.3	9.2–16.3(13.2)	12.0–15.6(14.0)
Body depth of anal fin origin	9.6	9.2–14.6(12.0)	11.3–15.4(12.9)
Pelvic fin origin to anus	25.5	19.3–25.9(22.9)	20.3–26.9(23.5)
Head depth	12.1	10.1–13.8(12.4)	11.3–14.2(13.4)
Percent head length (%)			
Snout length	21.1	19.4–30.1(24.6)	15.2–28.6(20.3)
Eye diameter	15.8	11.4–19.5(14.9)	13.1–21.5(17.1)
Cheek depth	23.2	15.2–28.4(24.1)	17.7–25.5(22.2)
Postorbital length	42.1	41.7–54.0(45.7)	45.7–58.2(49.3)
Lower jaw length	26.3	18.8–37.0(29.6)	15.3–25.5(22.7)
Interorbital width	33.7	25.9–39.3(32.4)	26.6–31.6(28.7)
Head width in maximum	49.5	43.3–65.5(54.9)	48.6–64.9(56.5)

##### Description.

***Fins***: The fin configuration includes 6 rays on the first dorsal fin (VI), with a 22 total rays. The second dorsal fin consists of one spine and either 9 or 10 branched rays, totaling 15 rays. The fourth or fifth spine of the first dorsal fin is the longest and non-filamentous. In males, when the first dorsal fin is depressed, the rear tip extends to the base of the second branched ray of the second dorsal fin, while in females it reaches only to the base of the second dorsal fin anteriorly. The anal fin has 1 spine and either 7 or 8 branched rays, totaling 13 rays. The origin of the anal fin is inserted at a vertical line between the first and second branched soft ray of the second dorsal fin. The pectoral fins range from 13 to 15 rays, with 13 rays most common (present in 8 specimens), 14 rays in 13 specimens, and 15 in 1 specimen. The pectoral fins are broad in shape.

***Scales***: The body covered with ctenoid scales, with enlarged mid-trunk scales. The anterior predorsal area lacks scales, while the posterior part of the occipital region is covered by cycloid scales. The belly is adorned with small cycloid scales. The longitudinal scale series count ranges from 30 to 33, with a mode of 31. The transverse scale series count ranges from 7 to 9, with a mode of 8.

***Head canals***: pores σ are located parallel to the anterior nares. The anterior interorbital sections of the oculoscapular canal are separated, featuring paired pore λ. There is a single pore κ in the posterior region, with ω present near posterior edge of eyes and a lack of ω1. The lateral section of anterior oculoscapular canal includes pores α and a terminal pore ρ. The posterior oculoscapular canal possesses two terminal pores θ and τ. Preopercular canals are presented, with pores ε, γ, and δ.

***Sensory papillae***: The sensory papillae arrangement is as follows: Row a extends to before the middle of the eye. Row b is oblique and reaches forward to the orbit. Rows c and d extend to the posterior margin of the eyes, and Row cp is absent. Row f is paired. In the opercular region, there are rows ot, oi, and os. Rows oi and ot are not connected.

***Vertebrae***: The total vertebrae count is 12 + 18 = 30 (*N* = 5) and (P–V) 3/II II I I 0/9 (*N* = 5).

***Coloration of preserved specimens***: In males, the head and body are gray with irregular markings on the cheeks and operculum. The ventral side is densely covered with tiny black spots and has six large, sometimes inconspicuous, horizontal black lines. The first dorsal fin is yellowish, While the second dorsal fin is yellowish-brown. The anal fin is yellowish. Females exhibit a gray head and body, with the first dorsal fin being yellowish and displaying blue spots between the 1^st^ and 2^nd^ spiny rays. The second dorsal fin is yellowish-brown, and the anal fin is yellowish.

**Figure 12.** Stream environment in Xiushan, Chongqing where *R.lithopolychroma* sp. nov. was collected.

***Colour in life***: Males display a light gray head and body with irregular orange markings on the cheeks and operculum, along with three smaller orange lines along the eyes. The ventral side is densely covered with tiny orange spots and has six large, sometimes inconspicuous, horizontal black lines. The first dorsal fin shows orange outlines on spines IV – VII with a white outer edge and blue spots between the 1^st^ and 2^nd^ spiny rays. The second dorsal fin is orange with irregular blue markings internally and on the outer edge, as well as blue spots on the 1^st^ and 2^nd^ spiny rays and a wide white margin. The anal fin is orange at the base, transitioning to black with a wide white margin. Females also exhibit a light gray head and body with irregular orange markings on the cheeks and operculum, and three smaller orange lines along the eyes. The ventral side is densely covered with tiny orange spots and features six large horizontal black lines. The first dorsal fin displays orange outlines on spines IV–VII with a yellow outer edge and blue spots between the 1^st^ and 2^nd^ spiny rays. The second dorsal fin is orange, and the anal fin is orange at the base, transitioning to black with a wide white margin.

##### Distribution and habitat.

*Rhinogobiuslithopolychroma* is restricted to fast-flowing, shallow streams with a cobble substrate in Xiushan, Chongqing. The surveyed streams ranged from 10 to 30 cm in depth. This goby species is characterized by its large eggs (1.5–2.1 mm in size), which it deposits on the bottom surface of the cobblestones.

##### Etymology.

*Rhinogobiuslithopolychroma* was discovered in a small stream with a colorful cobble substrate. Accordingly, we named this species after its habitat. In Ancient Greek, “litho” means “stone,” and “polychroma” means rich in color. We combined these two words to christen this species. We suggest the Chinese name of this species as “彩石吻虾虎鱼”.

## ﻿Discussion

*Rhinogobiussudoccidentalis* and *R.lithopolychroma* are found in close geographical proximity and share some environmental commonalities, yet their morphology differs considerably. *Rhinogobiussudoccidentalis* typically features a longitudinal scale series of 30–33, while *R.lithopolychroma* exhibits 22–24 scales. In body coloration, *R.sudoccidentalis* appears creamy white with black spots on the cheeks and operculum, and a densely spotted ventral side. Conversely, *R.lithopolychroma* is light gray with irregular orange markings on the cheeks and operculum, and a ventral side densely covered with tiny orange spots, often accompanied by six large, occasionally inconspicuous, horizontal lines of black.

**Figure 14.**
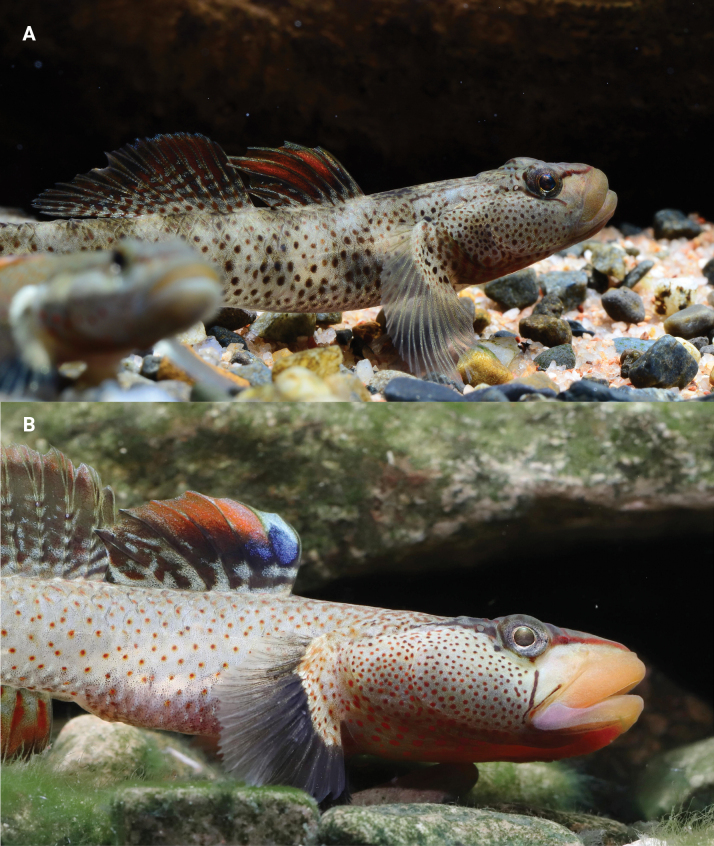
Pictures of *R.reticulatus* and *R.sudoccidentalis* sp. nov. with the latter having black lines under the eyes **A***R.reticulatus***B***R.sudoccidentalis*.

Morphologically, *R.sudoccidentalis* bears the closest resemblance to *Rhinogobiusreticulatus* Li, Zhong & Wu, 2007 (Fig. 14A, B). They can be distinguished from other *Rhinogobius* species by their similar creamy white body coloration, reddish-brown stripes on the snout, and densely spotted ventral sides. To differentiate *R.sudoccidentalis* from *R.reticulatus*, one should observe traits such as the absence of predorsal scales in *R.sudoccidentalis* compared 3–6 in *R.reticulatus*, and the presence of a lower jaw stripe absent in *R.reticulatus*. The closest morphological match to *R.lithopolychroma* is *R.cliffordpopei*. *Rhinogobiuslithopolychroma* and *R.cliffordpopei* share several distinguishing characteristics, including VI rays in the first dorsal fin, I,7–8 rays in the anal fin, and a predorsal scale series count of 0. They also exhibit similar body coloration. However, *R.lithopolychroma* differs from *R.cliffordpopei* in having 13–15 pectoral fin rays compared to 17–21 in *R.cliffordpopei*, and a total vertebrae count of 30 versus 26 in *R.cliffordpopei* (Li 2011).

**Figure 15.**
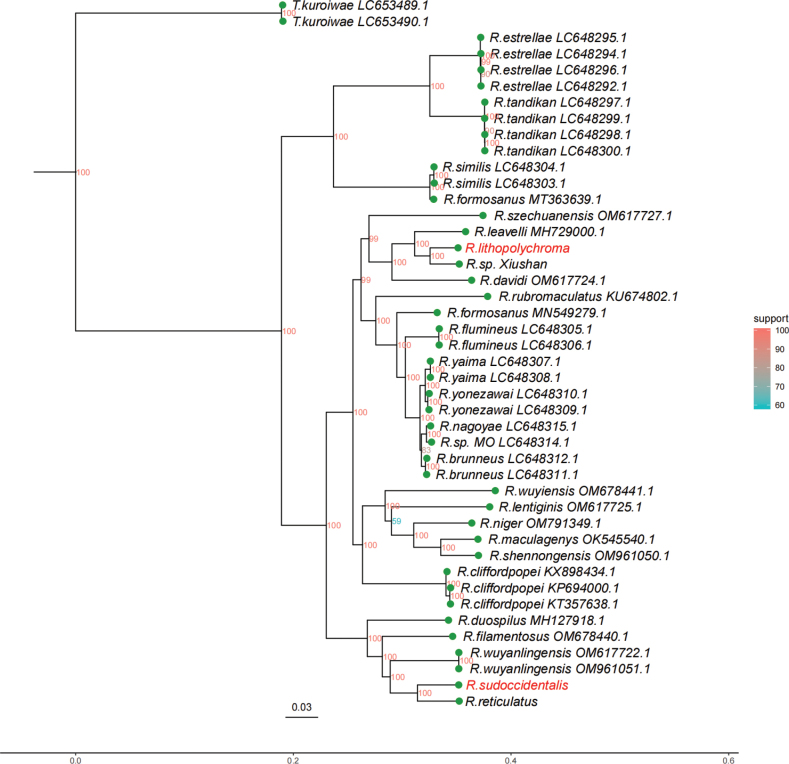
Maximum likelihood phylogenetic tree for *Rhinogobius* species having mitochondrial genomes sequences available, including the two new species highlighted in red.

As depicted in the phylogenetic tree, *R.lithopolychroma* is closest to *Rhinogobiusleavelli* (Herre, 1935) and *Rhinogobiusdavidi* (Sauvage & Dabry de Thiersant, 1874), whereas *R.sudoccidentalis* is closest to *Rhinogobiusfilamentosus* (Wu, 1939), *R.wuyanlingensis*, *R.reticulatus* and *Rhinogobiusduospilus* (Herre, 1935) (Fig. 15). *Rhinogobiuslithopolychroma* shares morphological similarities with *R.leavelli* and *R.davidi*, but distinguishes itself with a higher vertebrae count and a naked predorsal area (Table 4), setting it apart from these species. Notably, *R.sudoccidentalis* also exhibits a high vertebrae count compared to closely related *Rhinogobius* species, and similarly features a naked predorsal area and a lower count of longitudinal scale (Table 5). Akihito et al. (2000) suggest that vertebrae counts may correlate with *Rhinogobius* ecotypes, with species inhabiting continental streams and rivers often displaying higher vertebrae counts (Chen and Miller 2008; Wanghe et al. 2020). The present study supports this view, noting that *R.leavelli*, *R.davidi*, *R.filamentosus*, *R.wuyanlingensis*, *R.reticulatus* and *R.duospilus* are primarily found in coastal provinces of southern China (Wu et al. 2008), while both new species are located in inland China. These two new species represent further evidence of vertebral and environmental adaptations within the genus *Rhinogobius*.

**Table 4.** Morphological comparison of *Rhinogobiuslithopolychroma* with the genetically closest species.

Variable	*R.lithopolychroma*	*R.leavelli*	*R.davidi*
1^st^ dorsal fin	VI	VI	VI
2^nd^ dorsal fin	I 9-10	I 8-9	I 9-10
Anal fin	I 7-8	I 8-9	I 6-8
Pectoral fin	13-15	14-15	14-15
Longitudinal scale	30-33	28-34	30-32
Transverse scale	7-9	9-11	11-12
Predorsal scale	0	6-12	0-4
Total vertebrae	30	26	28
References	This study	Wu et al. 2008; Li 2011	Wu et al. 2008; Li 2011

**Table 5.** Morphological comparison of *Rhinogobiussudoccidentalis* with the genetically closest species.

Variable	*R.sudoccidentalis*	*R.filamentosus*	*R.wuyanlingensis*	*R.reticulatus*	*R.duospilus*
1^st^ dorsal fin	VI–VII	V–VI	V–VI	VI	VI
2^nd^ dorsal fin	I 8-9	I 8-9	I 8-9	I 8-9	I 8-9
Anal fin	I 6-7	I 8	I 8	I 7-8	I 6-7
Pectoral fin	14-15	15-17	17-18	15-17	15-16
Longitudinal scale	22-24	30-33	30-32	27-29	30-32
Transverse scale	7-8	8-10	9-10	8-9	8-10
Predorsal scale	0	5-11	7-9	3-6	6-10
Total vertebrae	29	27	27	26-27	27
References	This study	Wu et al. 2008	Huang et al. 2016	Li et al. 2007	Wu et al. 2008; Li 2011

According to studies by Yamasaki et al. (2015) and Li (2011) on *Rhinogobius* species, there is a correlation between egg size and species habitat preferences. Yamasaki defined small eggs as 0.6–0.9 mm and larger eggs as 1.4–2.1 mm. Li’s research in 2011, conducted in the Qiantang River, demonstrated that species like *R.duospilus* and *R.davidi* inhabited streams and produced large eggs, whereas *R.similis*, typically was found in pond reservoirs and produced small eggs. Yamasaki et al. (2015) further highlighted that species with small eggs generally have an amphidromous lifestyle (Takahashi and Yanagisawa 1999; Keith et al. 2015), while those with large eggs tend to exclusively inhabit streams.

In the Upper Youshui River catchment, previously documented *Rhinogobius* species include *R.similis* and *R.cliffordpopei*, known to favor lakes, reservoirs, and stagnant water environments. Conversely, the new species discovered in this study exclusively inhabit streams. These newly identified species are all classified as large-egg types, indicating their better adaptation to stream habitats compared to the small-egg types like *R.similis* and *R.cliffordpopei* (Li 2011). Furthermore, the four newly uncovered species exhibit distinct preferences within stream habitats. For instance, *R.lithopolychroma* thrives in environments characterized by strong currents and low temperatures, specifically alpine streams with chilly waters, where it represents the predominant *Rhinogobius* species. On the other hand, *R.sudoccidentalis* demonstrates a broader distribution and adaptability, being found in streams with warmer water temperatures, including urban streams. This diversity in habitat preferences suggests ecological niche differentiation, likely playing a pivotal role in the formation of *Rhinogobius* species.

Presently, the survival of the two recently discovered *Rhinogobius* species faces certain threats. For instance, manganese ore collection in the headwaters of streams where *R.sudoccidentalis* resides may have significant implications for the species survival. Additionally, *R.lithopolychroma* is restricted to a narrow habitat and is only found in alpine streams, underscoring the importance of prioritizing its protection and conducting further detailed studies on its biology and ecology.

## Supplementary Material

XML Treatment for Rhinogobius sudoccidentalis

XML Treatment for Rhinogobius lithopolychroma

## ﻿Additional information

### Author ORCIDs

Lingzhen Li https://orcid.org/0009-0008-2139-4009

Suxing Fu https://orcid.org/0009-0001-0562-1469

### Data availability

All of the data that support the findings of this study are available in the main text.
